# Supplementary material for: The Evolution and Characterization of the RNA Interference Pathways in Lophotrochozoa
Source: Genome Biol Evol. 2024 May 7;16(5):evae098. doi: 10.1093/gbe/evae098 (PMC11114477; doi:10.1093/gbe/evae098)
Supplement: evae098_Supplementary_Data [file evae098_supplementary_data.zip › Supplementary_materials.zip!/selected_miRNAs/NoGe_selected_miRNAs/mirdeep_pdf_files/NMRB01002591.1_Notospermus_geniculatus_strain_Ushimado_scaffold2591,_whole_genome_shotgun_sequence_39377.pdf]

Provisional ID	:	NMRB01002591.1_Notospermus_geniculatus_strain_Ushimado_scaffold2591,_whole_genome_shotgun_sequence_39377
Score total	:	1721
Score for star read(s)	:	3.9
Score for read counts	:	1712.1
Score for mfe	:	1.9
Score for randfold	:	0
Score for cons. seed	:	3
Total read count	:	3370
Mature read count	:	3164
Loop read count	:	0
Star read count	:	206

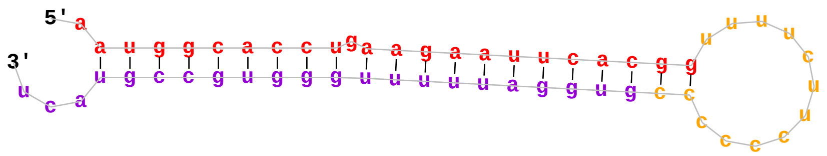

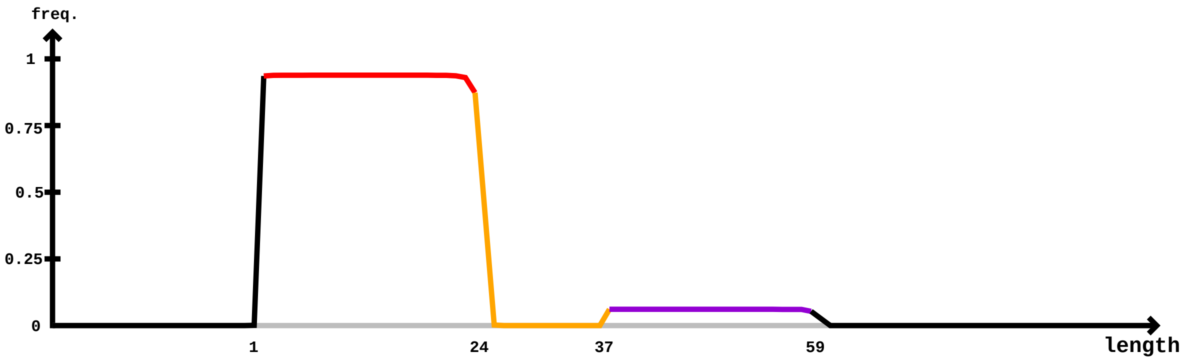

Mature **Star**

```
5'- gcuuugcuuuugucuccaagaauggcaccugaagaauucacgguuuucuuccccccguggauuuuugggugccguacuuggguugaugguuuuucaucacugagccauguuuc   -3'   obs
    gcuuugcuuuugucuccaagaauggcaccugaagaauucacgguuuucuuccccccguggauuuuugggugccguacuuggguugaugguuuuucaucacugagccauguuuc         exp
    ..............(((((.(((((((((.(((((((((((((...........))))))))))))))))))))).)))))...(((((((.........))))))).....   reads   mm   sample
    ...................Caauggcaccugaagaauucacgg......................................................................   2       1    Mm2
    ....................aauggcaccugaagaauu...........................................................................   1       0    Mm2
    ....................aauggcaccugaagaauuca.........................................................................   4       0    Mm2
    ....................aauggcaccugaagaauucac........................................................................   13      0    Mm2
    ....................aauggcaccugaagaauucacU.......................................................................   2       1    Mm2
    ....................Nauggcaccugaagaauucacg.......................................................................   1       1    Mm2
    ....................aauggcaccugaagaauucacg.......................................................................   101     0    Mm2
    ....................aauggUaccugaagaauucacgg......................................................................   2       1    Mm2
    ....................aauggcaAcugaagaauucacgg......................................................................   1       1    Mm2
    ....................aauggcaccugaagaaCucacgg......................................................................   7       1    Mm2
    ....................aauggcaccugaagaauucacgg......................................................................   2089    0    Mm2
    ....................aauggcaccugaagaauucCcgg......................................................................   1       1    Mm2
    ....................aauggcaccugaagaauCcacgg......................................................................   14      1    Mm2
    ....................aauggcaccuAaagaauucacgg......................................................................   2       1    Mm2
    ....................aauggcaccugaagaauucaUgg......................................................................   1       1    Mm2
    ....................aauggcaccugaagGauucacgg......................................................................   1       1    Mm2
    ....................aUuggcaccugaagaauucacgg......................................................................   1       1    Mm2
    ....................aauAgcaccugaagaauucacgg......................................................................   1       1    Mm2
    ....................aauggcaccugaagaaGucacgg......................................................................   4       1    Mm2
    ....................aauggcaccugaagaaAucacgg......................................................................   4       1    Mm2
    ....................aaugUcaccugaagaauucacgg......................................................................   2       1    Mm2
    ....................aauggcaccugaagaauucacgC......................................................................   5       1    Mm2
    ....................aauggcUccugaagaauucacgg......................................................................   1       1    Mm2
    ....................aauggcaccugaagaauAcacgg......................................................................   9       1    Mm2
    ....................aauggcaccCgaagaauucacgg......................................................................   2       1    Mm2
    ....................Uauggcaccugaagaauucacgg......................................................................   2       1    Mm2
    ....................aauggcaccugaagaauGcacgg......................................................................   14      1    Mm2
    ....................aauggcaccugaUgaauucacgg......................................................................   1       1    Mm2
    ....................aauCgcaccugaagaauucacgg......................................................................   1       1    Mm2
    ....................aauggcaccugaagaauucacgU......................................................................   1       1    Mm2
    ....................aaCggcaccugaagaauucacgg......................................................................   1       1    Mm2
    ....................aaAggcaccugaagaauucacgg......................................................................   1       1    Mm2
    ....................aauggcaccugaagaauucacgA......................................................................   9       1    Mm2
```

```
                    Mature                             Star
gcuuugcuuuugucuccaagaauggcaccugaagaauucacgguuuucuucccccguggauuuuugggugccguacuuggguugaugguuuuucaucacugagccauguuuc
....................aauggcaccuCaagaauucacgg.....................................................................   4     1   Mm2
....................aauggcaccugaagaauuUacgg.....................................................................   1     1   Mm2
....................aauggcaccugaagaauAcacggu....................................................................   2     1   Mm2
....................aauggcaccugaagaauucacggG....................................................................   1     1   Mm2
....................aauggcaccugaagaauucacggu....................................................................   13    0   Mm2
....................aauggcaccugaagaauGcacggu....................................................................   1     1   Mm2
....................aauggcaccugaagaauucacggA....................................................................   12    1   Mm2
....................aauggcaccugaagaauucacggAu...................................................................   1     1   Mm2
....................aauggcaccugaagaauucacgguu...................................................................   5     0   Mm2
.....................auggcaccugaagaauucacg......................................................................   2     0   Mm2
.....................auggcaccugaagaauucacgg.....................................................................   6     0   Mm2
......................uggcaccugaagaauucacgg.....................................................................   1     0   Mm2
.........................caccugaagaauucacgg.....................................................................   1     0   Mm2
.......................................................guggauuuuugggugccg.......................................   2     0   Mm2
.......................................................guggauuuuugggugccguac....................................   4     0   Mm2
.......................................................guggauuuuugggugccguacG...................................   1     1   Mm2
.......................................................guggauuuuugggugccguacu...................................   12    0   Mm2

....................aauggcaccugaagaauuca........................................................................   1     0   Mm1
....................aauggcaccugaagaauucac.......................................................................   2     0   Mm1
....................aauggcaccugaagaauucacg......................................................................   15    0   Mm1
....................aUuggcaccugaagaauucacg......................................................................   1     1   Mm1
....................aauggcaccugaagaauucacgg.....................................................................   116   0   Mm1
....................aauggcaccugaagaauucacgU.....................................................................   1     1   Mm1
....................aauggcaccugaagaaGucacgg.....................................................................   1     1   Mm1
....................aauggcaccugaagaauucacgA.....................................................................   5     1   Mm1
....................aauggcaccugaagaauAcacgg.....................................................................   2     1   Mm1
....................aauggcaccugaagaauucacggu....................................................................   4     0   Mm1

....................aauggcaccugaagaauucacg......................................................................   24    0   Fm1
....................aauAgcaccugaagaauucacgg.....................................................................   1     1   Fm1
....................aauggcaccugaagaauucacgg.....................................................................   94    0   Fm1
....................aauggcaccugaagaauAcacgg.....................................................................   1     1   Fm1
....................aauggcacUugaagaauucacgg.....................................................................   1     1   Fm1
....................aauggcaccugaagaauucacggu....................................................................   1     0   Fm1
....................aauggcaccugaagaauucacggA....................................................................   1     1   Fm1
....................aauggcaccugaagaauucacgguu...................................................................   1     0   Fm1
.......................................................Auggauuuuugggugccguac....................................   19    1   Fm1
.......................................................Auggauuuuugggugccguacu...................................   163   1   Fm1

....................aauggcaccugaagaauucacg......................................................................   10    0   Fm2
....................aauggcaccugaagaauAcacgg.....................................................................   1     1   Fm2
....................aauggcaccugaagaauGcacgg.....................................................................   3     1   Fm2
....................aauggcaccugaagaauCcacgg.....................................................................   2     1   Fm2
....................aauggcaccugUagaauucacgg.....................................................................   1     1   Fm2
....................aauggcaccugaagaauucaUgg.....................................................................   1     1   Fm2
....................aauggcacUugaagaauucacgg.....................................................................   1     1   Fm2
....................aauggcaccugaagaaGucacgg.....................................................................   1     1   Fm2
....................aauggcaccugaagaauucacgg.....................................................................   236   0   Fm2
....................aauggcaccugaagaauucacggu....................................................................   19    0   Fm2
....................aauggcaccugaagaauucacggA....................................................................   5     1   Fm2
....................aauggcaccugaagaauAcacggu....................................................................   1     1   Fm2

....................aauggcaccugaagaauuca........................................................................   1     0   Mm3
....................aauggcaccugaagaauucac.......................................................................   4     0   Mm3
....................aauggcaccugaagaauucacg......................................................................   13    0   Mm3
....................aauggcaccugaagaauucacgU.....................................................................   1     1   Mm3
....................aauggcaccugaagaauucacgg.....................................................................   174   0   Mm3
....................aauggUaccugaagaauucacgg.....................................................................   2     1   Mm3
....................aauggcaccugaagaauucacgA.....................................................................   4     1   Mm3
....................aauggcaccugaagaauCcacgg.....................................................................   1     1   Mm3
....................aauggcaccugaagaauGcacgg.....................................................................   1     1   Mm3
....................aauggcaccugaagaauucacggu....................................................................   1     0   Mm3
.......................................................guggauuuuugggugccguac....................................   1     0   Mm3
.......................................................guggauuuuugggugccguacu...................................   4     0   Mm3

...................Caauggcaccugaagaauucacgg.....................................................................   1     1   Fm3
....................aauggcaccugaagaauu..........................................................................   1     0   Fm3
....................aauggcaccugaagaauucac.......................................................................   3     0   Fm3
....................aauggcaccugaagaauucacg......................................................................   22    0   Fm3
```

```
                    Mature                               Star
gcuuugcuuuugucuccaagaauggcaccugaagaauucacgguuuucuucccccguggauuuuugggugccguacuuggguugaugguuuuucaucacugagccauguuuc
....................aauggcaccugaagaauucacgg.......................................................................    29          0          Fm3
....................aauggcaccugaagaaCucacgg.......................................................................    1           1          Fm3
....................aauggcaccugUagaauucacggu......................................................................    1           1          Fm3
```
